# Mechanisms associated with maternal adverse childhood experiences on offspring’s mental health in Nairobi informal settlements: a mediational model testing approach

**DOI:** 10.1186/s12888-018-1953-y

**Published:** 2018-12-05

**Authors:** Manasi Kumar, Beatrice Amugune, Beatrice Madeghe, Grace Nduku Wambua, Judith Osok, Anastasia Polkonikova-Wamoto, David Bukusi, Fred Were, Keng-Yen Huang

**Affiliations:** 1 Department of Psychiatry, University of Nairobi, Nairobi, Kenya; 2 Research Department of Clinical Health and Educational Psychology, University College London, London, UK; 3 Department of Pharmacy, University of Nairobi, Nairobi, Kenya; 4 Department of Food and Nutrition Sciences, University of Nairobi, Nairobi, Kenya; 5 Department of Clinical, Neuro- & Developmental Psychology, Vrije University of Amsterdam, Amsterdam, Netherlands; 6 Head VCT and HIV Care, Kenyatta National Hospital, Nairobi, Kenya; 7 College of Health Sciences, University of Nairobi, Nairobi, Kenya; 8 Department of Population Health, New York University School of Medicine, New York, USA

**Keywords:** Maternal mental health, Adverse childhood experiences, Family stress model, Internalizing problems, Externalizing problems, Urban poverty

## Abstract

**Background:**

Adverse childhood experiences (ACEs) is a significant public health and social welfare problem in low-and middle income countries (LMICs). However, most ACEs research is based on developed countries, and little is known about mechanisms of early ACEs on adulthood health and offspring’s wellbeing for populations in LMICs. This area is needed to guide social welfare policy and intervention service planning. This study addresses these research gaps by examining patterns of ACEs and understanding the role of ACEs on adulthood health (i.e., physical, mental health, experience of underage pregnancy) and offspring’s mental health in Kenya. The study was guided by an Integrated Family Stress and Adverse Childhood Experiences Mediation Framework.

**Methods:**

Three hundred ninety four mothers from two informal communities in Kariobangi and Kangemi in Nairobi were included in this study. The Adverse Childhood Experiences International Questionnaire (ACE-IQ), the Kessler Psychological Distress Scale (K10), Overall Health and Quality of Life items, and Child Behavior Checklist were used to study research questions. Data was gathered through a one-time interview with mothers. Structural Equational Modeling (SEM) was applied for mediational mechanism testing.

**Results:**

Among 13 ACE areas, most mothers experienced multiple adversity during their childhood (Mean (SD) = 4.93 (2.52)), with household member treated violently (75%) as the most common ACE. SEM results showedthat all domains of ACEs were associated with some aspects of maternal health, and all three domains of maternal health (maternal mental health, physical health, and adolescent pregnancy) were significantly associated with development of offspring’s mental health problems.

**Conclusion:**

ACEs are highly prevalent in Kenyan informal settlements. Consistent with cross cultural literature on family stress model, maternal ACEs are robust predictors for poor child mental health. Preventive interventions for child mental health need to address maternal adverse childhood traumatic experiences as well as their current health in order to effectively promote child mental health.

## Background

In Kenya, it is estimated that 60 to 80% of urban residents live in informal settlements with slum like conditions [1]. More than half of the estimated population of 46 million Kenyans are young people under 18 of years of age [2]. A large number of those grow up experiencing poverty, poor health, nutrition, and deficient care [3] According to World Bank, 36.1% of the Kenyan population lives below the poverty line [4]. This population comprises of 52.3% in the rural and 34.8% of urban dwellers living in poverty [1]. Between 60 and 70% of Nairobi city’s population live in congested informal settlements, commonly referred to as slums, without proper access to sanitation, clean water, health care and other social services [5].

Adversity and stressful environment can lead to parental depression, which has a detrimental impact on parenting, family functioning, parent-child relationships, and offspring’s physical, social and behavioral health, and cognitive functioning [6]. According to findings of the National survey on violence against children, (KVACS, 2010), child abuse and neglect are common adversity events experienced by many Kenyan caregivers during their childhood. KVACS report on adults further indicates that during childhood, 32% of females and 18% of males experience sexual violence, 66% of females and 73% of males’ experienced physical violence, 26% of females and 32% of males experience any violence as a child and 13% of females and 9% of males experienced all three types of violence during childhood [7]. Similarly, Mbagaya, Oburu & Bakermans-Kranenburg [8] reported 59% of child physical abuse (without differentiation of its severity in Kenya), and most violence against children was perpetuated by parents or close relatives [7].

There is paucity of new data to authoritatively evaluate long term impacts of ACEs on adulthood wellbeing and offspring’s health and development in Kenya. This knowledge gap also exists for other Sub-Saharan African (SSA) countries. In SSA context, it is likely that the effects of poverty and limited resources would exacerbate the impact of adverse childhood experiences on adulthood and their offspring’s health. This study aims to address these gaps by examining the relationship between adverse childhood experiences and their offspring’s mental health.

### Our integrated conceptual framework

This study was guided by the integration of the Family Stress Model [9–12] and the adverse childhood experiences (ACE) framework, proposed by the Centre for Disease Control/World Health Organization [13, 14] which posits that the level of parental and environmental-related stress influences family functioning and child outcomes. The Family Stress model specifically focuses on the current experience of severe economic pressures and other stressors that tend to undermine parents’ mental health, family functioning and subsequent child adjustment [15]. The ACE framework focuses on how early experience of neglect, psychological distress and violence related stressors impacts on an individual’s health-risk behaviors and adulthood chronic disease development. This framework focuses more on the impact on the individual’s long-term outcomes, and less on their impact on the offspring. There are a couple of empirical findings about ACE which are worth noting here. *ACEs are common across cultures and social class contexts* with higher vulnerability to some groups such as those in economic hardships or refugee contexts. *ACEs form into clusters* therefore when analyzing their impact and addressing these it is important to see their *cumulative impact* than individual impact. What is of most relevance is that *ACEs have strongly demonstrated a dose-response relationship* with adult psychopathology as well as with other health conditions [16]. Some studies have argued that whilst ACEs were significantly predictive of all adult mental health outcomes beyond demographic and socioeconomic characteristics; social disadvantage continues to be identified as being a unique contributing factor and therefore merits continued examination of ACEs integrated within a social disadvantage framework [17, 18]. This study utilizes an integrated ACEs and family stress model, that allows us to examine impact of ACEs on adolescent health-risky behaviors (i.e., adolescent pregnancy) and adulthood health (i.e., physical and mental health), and influence of these current family contexts on offspring’s health and development.

## Objectives and associated hypotheses

Guided by the integrated ACEs and Family Stress framework, this study included four research objectives.

1. Describe patterns of ACEs in 13 areas among Kenyan mothers from two vulnerable communities located within Nairobi informal settlements in Kenya.
*We hypothesize high level of ACEs would be found in women who live in informal settlements.*
2. Examine whether mothers’ ACEs associated with adverse health outcomes in adulthood (including physical health, mental health, and adolescent pregnancy).
*We hypothesize ACEs experienced in maternal caregivers’ childhood would negatively associated with their adulthood functioning.*
3. Examine whether ACEs related to offspring’s mental health problems.
*We hypothesize maternal ACE exposure would be significant predictors of greater offspring internalizing and externalizing problems.*

## Methods

### Participants

The original sample included 431 parents (415 mothers and 16 fathers). Parents/primary caregivers who had 2–16 years old children and seeking maternal and child health services at Kariobangi and Kangemi public health centers were eligible to the study. Parents in this study were defined as biological birth parents. Parents who were psychologically unstable were ineligible for the study. For the purpose of this study, we further limited our participants to mothers (*n* = 415) and focused on the psychopathology mechanism testing in the family context where mothers are the primary caregivers. We excluded fathers given the small sample size (*n* = 16). Among the eligible mothers, 394 (95%) had data for all study variables. They were considered as the final analytical sample for this study.

The original study sample size was estimated based on the anticipated small to moderate effect *f*^*2*^ = .04 to .10 (or R^2^ = 4–16%), 10 observed variables (8 independent variables, 2 dependent variables), with power level .80, and probability level .05 of a SEM model. We anticipate a minimum sample size of 160–380 would be required [19].

### Setting and data collection procedure

Participants were recruited between Oct 2015 to April 2016 from two maternal and child health (MCH) centers from two underserved informal settlement communities of Nairobi. We chose these health service settings because families of young children tend to utilize preventive or treatment services from these providers. Eligible parents (describe above) were approached to obtain oral or written informed consent. For parents who were literate, the consent information was shared verbally, and a written consent form (with written information about the study) was given at the time of obtaining consent. A signed consent form was documented for parents who agreed to participate. For parents who were illiterate, an oral consent was obtained, and a literate witness (e.g., an adult who parents trusted) signed the consent form on their behalf, which was then documented. Consent and interviews were conducted in Kiswahili (99%; as it is the most common local language in Kenya) or English (1%, the official language in Kenya education system) based on parents’ preference.

After consent, parents were scheduled for an interview at the MCH center by a trained bachelor or masters-level social science researcher. Participating parents were asked questions related to their childhood experience, their current health and wellbeing, and their child’s health. For families with more than one child (age range 2–16 years), only one child was targeted. Children did not directly participate in the study activities, and they were not the subjects of this study. The study procedures and method of consent was approved by the Institutional Review Board of the Kenyatta National Hospital/University of Nairobi (IRB number: KNH/UoN-ERC Ref. P520/08/2015). To comply with ethical guidelines, participants who identified with a high depression score (cut-off of 10 or more based on our interview questionnaire Kessler Psychological Distress Scale; described below) or requested for other mental health services (due to stress or anxiety) during the data collection interview were referred to the Mental Health Clinic or provided with some onsite psychosocial support.

### Measures

All study measures, except CBCL and parental health, have been used in Kenyan context, and are available in Kiswahili. To validate CBCL and parental health, a formal translation process was undertaken with permission from the test-developer Achenbach. The Kiswahili version of questionnaires was translated and back-translated, and a team review approach was used to resolve any discrepancies between the versions. Scale reliability (assessed using internal consistency Cronbach’s Alpha/α) and validity for all study measures based on current study sample are reported below, which demonstrated all study measures to be reliable and valid with our study Kenya sample.

#### Adverse childhood experiences

Was assessed using *The Adverse Childhood Experiences International Questionnaire (ACE-IQ)* [20]. ACE-IQ assesses adult caregivers’ experiences of childhood adversities such as child maltreatment and other traumatic stressors. Caregivers were asked to rate the frequency (never to many times/always) of their childhood experiences on 29 items, which were then categorized and converted to yes/no binary score in 13 areas. These binary scores were used to estimate prevalence of ACEs in diverse areas. To capture dose/level of the ACEs, summary scores (sum score) in 5 domains as well as a total score (sum of 13 areas) were also created. These include *Neglect domain* (2 ACE areas), *Family Psychological Distress domain* (4 ACE areas), *Home Violence domain* (3 ACE areas), *Sexual violence* (1 ACE area), and *Community Violence domain* (3 ACE areas) (see Table 1 for ACE areas and domains). These summary scores were used for subsequent psychopathology analyses. Inter-correlations (*r*) among four ACE domain scores were raged from .16–.26 (*p* < .001).

**Table 1:** Caregivers’ Adverse Childhood Experiences

ACE (Yes/No in 13 areas)	Examples (number of items)	Prevalence
*Neglect*		%
1 Physical neglect	1. Caregiver not give you enough food or sending you to school even it could easily been done; too drunk/ intoxicated by drugs to take care of you (any 3 items)	38.5
2 Emotion neglect	2. Caregiver not understand your problems/worries, knowing what you were doing with your free time (any 2 items)	42.6
*Psychological Distress of Family Members*		
3 One or no parents, parental separation or divorce	1. Parents ever separated/divorced, or father/mother/guardian die (any 2 items)	53.8
4 Alcohol/drug abuser in the household	2. Live with a household member who was a problem drinker, alcoholic, or misused street/prescription drugs (1 item)	44.1
5 Someone chronically depressed, mentally ill	3. Live with a household member who was depressed, mentally ill, or suicidal (1 item)	21.7
6 Incarcerated household member	4. Live with a household member who ever sent to jail or prison (1 item)	19.6
*Violence at Home*		
7 Household member treated violently	1. See or hear a parent/household member being yelled at/screamed at/sworn at/ insulted or humiliated; being slapped/kicked/punched/beaten up; or being hit/cut with an object (any 3 items)	74.8
8 Emotion abuse	2. You being yelled/screamed/sworn/insulted or humiliated, or being threaten/abandon, thrown out by parents/guardian (any 2 items)	38.0
9 Physical abuse	3. You being spanked/ slapped/kicked/ punched/beaten up; or being hit/cut with an object by parents/guardian (any 2 items)	39.2
*Violence in Community*		
10 Bullying many time	1. Being bullied (1 item)	22.4
11 Community violence	2. See or hear someone being beaten up, stabbed, short, or being threatened with a knife or gun in real life (any 3 items)	44.6
12 Collective violence	3. Force to go and live in other place; family member or friend killed or beaten up by soldiers, police, gangs, or militia (any 4 items)	39.6
*Sexual violence*		
13 Contact sexual abuse	1. Someone touch/fondle you, make you touch their body, attempt oral/anal/vaginal intercourse with you, or actually have intercourse with you in a sexual way when you did not want them to (any 4 item)	16.0
*Nnumber of ACE Risk By Domain*		Mean (SD)
Neglect (score 0–2)	• Physical + Emotional Neglect	0.81 (0.72)
Psychological distress of family members (score 0–4)	• One or no parent + substance abuse + mental ill + incarcerated household member	1.38 (1.09)
Violence at home (score 0–3)	• Emotion abuse + physical abuse + household member treated violently	1.52 (1.04)
Violence in community (score 0–3)	• Bullying + community + collective violence	1.06 (0.90)
*Total # ACE risk (0–13)*	13 areas in ACEs	4.93 (2.52)

#### Maternal Physical and Mental Health

Three self-report measures were used to assess maternal physical and mental health. The *Kessler Psychological Distress Scale* (K10; 10 items, α = .94 based on the current study sample) [21] assessed anxiety and depressive symptoms that mothers experienced. Mothers rated 10 symptom items on a 5-point scale (1 = none of the time; 5 = all of the time). A total score was created for 10 symptom items. Based on the recommendation, a clinical cut-off score of 25 was also used to estimate prevalence for mental disorder Individuals with a score of 25 or above would suggest a high likelihood of having a mental disorder. The scale has demonstrated to have good predictive validity using Structured Clinical Interview for DSM-IV (SCID) [22]. *Parental health* was assessed based on mother perception of overall health and quality of life (2 items; α = .70 for the study sample) on a 5-point scale (1 = poor, 2 = fair, 3 = good, 4 = very good, 5 = excellent) [23]). A mean score ≤ 2 indicates poor health. *Adolescent pregnancy status* (1 = give birth to the first child at age 18 or younger, 0 = give birth to the first child ≥19 years) was also used to estimate adolescent risky health behavior. Inter-correlation (*r*) between maternal mental health (K10) and maternal health were 0.34, and between adolescent pregnancy and mental health were 0.16 (all with *ps* < .05), suggesting sound validity of the measures for our Kenyan sample.

#### Child Mental Health

CBCL was used to assess child internalizing and externalizing problems on a 3-point scale (0 = not true, 2 = very or often true). Internalizing disorders present with an inward directed emotional and behavioral distress such as depression and externalizing disorders present with a propensity towards expressing distress outwards such as conduct disorder. Two CBCL age versions and 3 norm samples were used to score standardized T scores (1.5–5 version, 6–18 version for 6–11 years old and for 11–18 years old [24]. The research team carried out a formal translation of the 6–18 years with permission from the CBCL author Thomas Achenbach. Scale reliability were adequate for both internalizing problem (α = .76–.85 based on our Kenyan 3 age-group samples) and externalizing problem (α = .87–.89 based on the current Kenyan samples).

#### Demographics and Covariates

To consider potential confounders, maternal age, education status (1 = secondary or higher, 0 = primary or less), marital status (1 = married/living with the partner, 0 = divorced/single/ widowed) and employed status (1 = employed/self-employed, 0 = unemployed/homemaker) were included. Food insecurity (using Household Hunger Scale; 3 items; if any item was rated as yes was considered as food insecure [25] was also included as a demographic indicator.

## Data analysis

To study *prevalence and patterns of ACEs*, a series of descriptive analyses were conducted. To study the *association between ACEs and maternal health* (physical, mental health, and adolescent pregnancy) *and child mental health* (i.e., externalizing such as aggression and conduct disorders and internalizing problems such as depression, anxiety and somatic complaints), a series of univariate logistic regression analyses were conducted. We examined impacts of 13 categories of ACEs separately to understand unique contribution to maternal and child health. In addition, to study “dose/level” of ACEs, we also examined four domains and total number of ACEs as predictors. To study *the mediational mechanism (ACEs ➔ maternal health ➔ child mental health),* we applied structural equation modeling (SEM), allowing for (a) meditational links from ACE domains to maternal health to child mental health, (b) variables within each domain (i.e., ACEs, maternal health, child mental health) to be correlated; and (c) adjusting for potential confounders (i.e., maternal age, education status, marital status, and employed status). The SEM model was tested using MPLUS 7] and used weighted least square mean and variance (MLSMV) estimation method. To judge the closeness of fit of the hypothesized association model, three indices were used [23]: chi square (χ^2^ > .05), root mean square error of approximation (RMSEA < .08), and comparative fit index (CFI > .95).

## Results

### Socio-demographic characteristics

Our sample comprises of 394 biological mothers with mean age of 31.56 years (SD = 7.06 years, 52% were < 30 years). About one half of the mothers (52%) in our sample were employed or categorized themselves as self-employed, 70% had educational attainment of primary school or less, 66% were married or living with partner, 32% had her 1st child at or before age 18 (or 60% at or before age 20). Most families (98%) were Christian, 63% experiencing food insecurity, and on average, had 2.84 children (SD = 1.33) and 4.82 (SD = 1.56) household members at home. Target children were on average 6.78 years old (SD = 3.04 years, 41% 1.5–5 years, 53% 6–11 years, and 6% 12–18 years), and 45% were boys.

### Adverse childhood experiences (ACEs) of mothers

Table 1 shows prevalence of adverse childhood experiences in 13 areas, and total numbers of ACE by domains and in all 13 areas. The average number of ACEs experienced by mothers was 4.93 (SD = 2.52). Among the 13 ACE areas, the categories of household member treated violently (75%), presence of one or no parents, parental separation or divorce or living with one or no parents (54%), community violence (45%) living with alcohol/drug abuser (44%), and emotion neglect (43%), and collective violence experience (40%) were the most common childhood adversity experiences.

### Association between maternal ACEs and maternal and child health

We first examined patterns of mental health of our study caregivers and their children. For maternal health, 33% of mothers had moderate to severe mental disorder indication based on K10 (using the clinical cut-off 25). Another striking finding was that 32% had their first child at the age of 18 years or before, and 42% reported being in poor or fair health. With regards to the offspring, 15.6 and 10.2% were at-risk for internalizing and externalizing problems (T score ≥ 60), respectively. For the purpose of the analysis, we also used T-score cutoff of 50 (above norm mean) (24), which resulted in 44% of children with the internalizing score above mean standardized T score, and 31% with the externalizing score above mean standardized T score.

Next, we examined the associations between ACEs and maternal and offspring’s health using logistic regression analyses. Univariate un-adjusted associations are reported in Table 2.

**Table 2:** Associations between ACEs and Maternal and Offspring’s Health

	Child Health				Maternal Health					
ACE (Yes/No in 13 areas)	At-risk for Child Externalizing Problems		At-Risk for Child Internalizing Problems		At-Risk for Maternal Mental Disorder		Adolescent Pregnancy		Poor Maternal Physical Health	
	OR	p	OR	p	OR	p	OR	p	OR	p
*Neglect*										
Physical neglect	1.56	.040	1.92	.002	3.33	<.001	1.83	.006	1.63	.017
Emotion neglect	0.65	.054	0.92	.685	1.22	.343	2.03	.001	1.36	.129
*Psychological Distress of Family Members*										
One or no parents, parental separation or divorce	1.07	.742	1.36	.128	1.17	.470	1.55	.045	1.01	.968
Alcohol/drug abuser in the household	1.03	.910	1.21	.338	1.37	.141	1.32	.205	1.15	.502
Someone chronically depressed, mentally ill	0.67	.147	0.66	.091	1.65	.042	1.18	.530	1.00	.983
Incarcerated household member	1.33	.275	1.17	.521	1.69	.040	1.14	.626	.913	.720
*Violence at Home*										
Household member treated violently	1.26	.357	1.85	.009	1.27	.334	1.18	.508	.840	.444
Emotion abuse	1.89	.003	4.14	<.001	2.36	<.001	2.34	<.001	1.49	.051
Physical abuse	2.32	<.001	3.37	<.001	1.81	.005	1.30	.232	1.15	.487
*Violence in Community*										
Bullying many time	1.44	.148	1.22	.411	1.95	.005	1.72	.029	2.00	.004
Community violence	1.68	.015	1.46	.061	1.14	.547	1.29	.241	1.07	.727
Collective violence	1.01	.951	1.18	.418	2.39	<.001	1.25	.306	1.08	.715
*Sexual violence*										
Contact sexual abuse	1.50	.145	1.49	.142	1.42	.205	1.93	.017	1.37	.243
*Number of ACE Risk By Domain* (Score Range)										
Neglect (0–2)	1.29	.065	1.01	.951	1.92	<.001	1.86	<.001	1.45	.008
Psychological distress of family members (0–4)	1.09	.373	1.02	.826	1.28	.010	1.22	.045	1.01	.923
Violence at home (0–3)	2.05	<.001	1.45	<.001	1.45	<.001	1.33	.008	1.09	.358
Violence in community (0–3)	1.23	.061	1.27	.044	1.55	<.001	1.30	.027	1.21	.084
*Total # ACE risk (0–13)*	1.21	<.001	1.11	.013	1.26	<.001	1.20	<.001	1.08	.051

*Note.* Analyses were based on univariate logistic regression

As hypothesized, all total number of risk in 5 ACE domains and across all ACE risks were associated with poor maternal mental health and early motherhood (being adolescent mothers). The odds for poor maternal health increase as number of ACEs increase. In addition, the most consistent ACE predictor for poor maternal health was emotional abuse and physical neglect. For child mental health, maternal experience of physical neglect, emotion abuse, and physical abuse during their childhood were most consistent predictors for poor offspring’s mental health. In addition, two ACE domains which were, *violence at home and violence in community,* and total number of ACE risks were significantly associated with poor child mental health (on both externalizing and internalizing problems).

### Mediational mechanisms

**Fig. 1:**
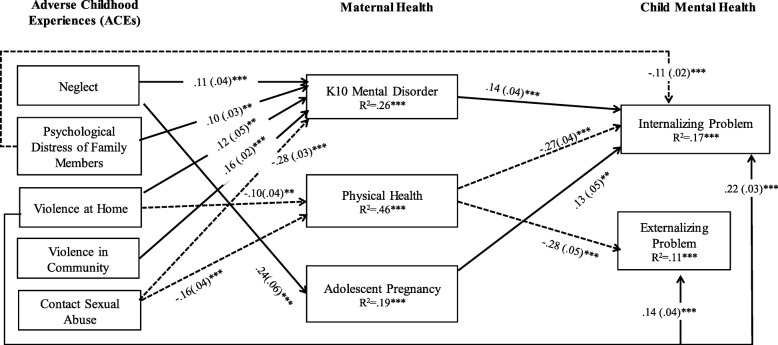
Structural Equation Modeling for Association among ACEs, Maternal Health, and Child Mental Health . *Note*. Maternal mental and physical health and child mental health variables were continuous variables. Adolescent pregnancy was a dichotomized variable (1=underage pregnancy). Model controlled for maternal age, education status, marital status, and employed status. Standardized path coefficients and standard errors (in parentheses) are presented. A dash path indicates a negative association. Paths that did not reach significance are not shown in this figure. * *p* < .05 ** *p* < .01 *** *p* < .001. Inter-correlations (*r*) among ACE variables ranged from .07 to .26, among maternal health variables ranged from .09-.31, and between child outcomes was .61

To examine whether the influence of ACEs on offspring’s mental health is mediated by caregivers’ health, adjusted SEM was carried out. Four demographic covariates (i.e., maternal age, education status, marital and employed status) were considered to adjust for potential confounders The overall χ^2^ statistics based on the original theoretical model showed a reasonable fit in the SEM, χ^2^ (10) = 59.69, p = <.001, RMSEA = .113 and CFI = .95. To improve the model fit, we add additional three paths (i.e., psychological distress of family member ➔ internalizing problem; violence at home ➔ externalizing and internalizing problems) guided by modification indexes suggested in the MPLUS. After including additional three paths, the overall χ^2^ statistics showed a better fit of the model, χ^2^ (7) = 24.16, p = <.001, RMSEA = .077 and CFI = .98. Figure 1 presents findings from this modified model. The standardized path coefficients for the significant paths and the *R*^*2*^ values for each endogenous variable (maternal health and child mental health variables) from the SEM are presented in the Figure.

After adjusting for demographic confounders, all 5 domains of ACEs were associated with some aspects of maternal health, and all maternal mental health and physical health were significantly associated development of child mental health problems. Specifically, we found expected mediation links that higher ACEs (i.e., neglect, psychological distress of family members, violence at home, violence in community) were associated with higher adulthood mental disorders, which further associated with higher offspring’s internalizing problem. In addition, exposure to violence at home and sexual abuse during childhood were associated with poor adulthood physical health, which further associated with more offspring internalizing and externalizing problems. Furthermore, higher level of neglect during childhood was associated with increase likely to have underage pregnancy, which further associated with higher offspring’s internalizing problem.

There were also two unexpected findings that we found childhood sexual abuse experience was associated with lower adulthood mental disorder, and experience with psychological distress of family members was associated with fewer offspring’s internalizing problem.

## Discussion

Our findings have several implications for maternal and child mental health. Our maternal caregivers had experienced adversities covering on an average five ACEs risk areas (out of 13 risk areas). Violence experienced at home was the most prevalent ACE followed by experience of psychological distress associated with family or caregivers. Both these domains imply a high level of interpersonal violence and traumatization experience that can lead to mental health problems in adulthood. Given that 50 % of our sample had experienced early motherhood and got pregnant as adolescents, we can see that their developmental trajectory was impacted in significant ways. Early pregnancy and motherhood in the Kenyan context are often associated with dysfunctional families, high poverty and violence exposure [26–29]. The odds for poor maternal health increase as number of ACEs increase was another critical finding of our work implying that the ACEs have a bearing on the current functioning of adult caregivers. A number of other studies have found that childhood adversity links to a wide range of health problems in adulthood [30]. Early pregnancy and motherhood are known adverse experiences for women that was later added in the expanded ACE questionnaire by WHO.

### Importance of ACEs in understanding adult well-being and child development

A number of ACE factors such as physical and emotional abuse and neglect, sexual abuse and parental separation or divorce or orphan-hood have been associated with pregnancy and motherhood experience in adolescence. These factors have been well-articulated in the literature on risk factors leading towards adolescent motherhood [31]. In our study we also found that in addition to ACEs predicting poor maternal mental health, other experiences such as physical neglect, emotional abuse and bullying were contributory factors. A number of studies have shown that children exposed to maternal depression are at risk for a wide range of negative cognitive, emotional and behavioral outcomes [32, 33]. Internalizing and externalizing problems are both implicated when caregivers have been through adversities and currently experience mental health problems. Overall all four ACE domains were significantly implicated with development of problem behaviors in the children as found in our analyses. While by and large a number of overlapping ACE domains were common to development of internalizing and externalizing problems, studies have shown that the level of caregiver depressive symptoms may also change over time and result in different trajectories of parenting behaviors [32, 34]. We did not assess changes longitudinally but perhaps a future study would take this into account. This may explain that some may lead to internalizing and others to externalizing problems. We also found that current mental health problems mediated the relationship between maternal ACEs and development of child mental health problems.

### Exposure to violence at home and community has implications for development of depression in maternal caregivers

Exposure to violence and interpersonal trauma in childhood is a precursor for depression and mental health problems in adulthood [35]. Given that our female participants experienced variable and protracted adversities, such as high stress, anxiety and food insecurity, the K10 scores indicate that there may be other comorbidities impacting their functioning. In another study where such relationship was explored the chronic life events course, implies more severe depression symptoms, and that would mean less recovery as compared to when there is depression only [36].

There are several limitations of the study. The ACE data were collected using retrospective data collection method, we relied on caregivers’ memory, which may not fully exclude recall bias. In addition, the assessment for maternal health and child mental health were concurrent assessments. Future research needs to include longitudinal data to better understand the causal mechanisms. This study was also limited to a maternal sample. Future studies should include fathers to better understand overall familial adversity experience on family functioning and offspring’s health and development. In such studies, ways of engaging fathers would need to be identified as caregiving is largely left to mothers in the Kenyan context. Finally, our sample was drawn from a peri-urban informal settlement and there may be variable patterns in rural poor or more geographically diverse pockets in Kenya. To have broader generalizability, further studies would need to include diverse populations.

### Clinical implications

Our study findings have several implications for clinical service development and intervention adaptation or engagement strategies development for women in Kenya. *Women with high adversities and stress need greater support to prevent intergenerational transmission of mental illness.* The adversity and stress experience of Kenyan women living in informal settlements has been well-documented. Interventions such as Problem Management Plus have been developed and championed by WHO to address adversity experiences and to bolster social support and treat depression in vulnerable women [37]. Low dose interventions delivered by community health workers or trained parent peer who may be able to factor in experience of adversities and ongoing depression and psychosocial functioning would be promising for women represented in our sample.

Similarly, a number of interventions focusing on maternal functioning have found positive impact on offspring well-being in recent times [38]. Parent peers can be a potential resource to deliver evidence-based psychosocial interventions [39]. Recent literature shows that ACEs could feed into more trauma-and-stress-mitigation informed practices and when this is combined with intergenerational approaches a number of interventions in clinical, early care, educational, and community contexts could be developed [40]. The especial focus on resilience and coping has been championed with ACE framework [41]. Parenting interventions targeting prosocial parenting and behavior development in families living in resource constraint and multiple adversity context would be helpful [42].

## Conclusion

Adverse childhood experiences are highly prevalent in the lived experience of adult caregivers based in Kenyan informal settlements. Our study findings also support the integrated ACE and Family Stress model that maternal ACEs being a strong predictor for poor maternal health and child mental health. Preventive interventions for child mental health would benefit if consideration was given to understanding more deeply entrenched maternal trauma and adversities so that their impact on parenting and child health outcomes could be addressed. Interventions targeting poor health or mental health of women living in low resource settings need to target adverse childhood experiences of adult women in addition to their ongoing stress to provide the right psychosocial and mental health support. To have more generalizable causal influence, future studies need to utilize longitudinal design, and include mothers and fathers from diverse regions. Future studies need to also include other mental disorders (e.g., trauma, conduct disorders) in addition to anxiety and depression disorders to better understand impacts of ACEs on adulthood mental health.

## Abbreviations

ACE: Adverse Childhood Experiences
ACE-IQ: Adverse Childhood Experiences International Questionnaire
CBCL: Child Behavior Checklist
K10: Kessler Psychological Distress Scale Overall Health and Quality of Life items
KVACS: Kenya Violence against Children Survey
MCH: Maternal and Child Health
SEM: Structural Equational Modeling
SSA: Sub-Saharan Africa
WHO: World Health Organization
